# COS-STAR: a reporting guideline for studies developing core outcome sets (protocol)

**DOI:** 10.1186/s13063-015-0913-9

**Published:** 2015-08-22

**Authors:** Jamie J. Kirkham, Sarah Gorst, Douglas G. Altman, Jane Blazeby, Mike Clarke, Declan Devane, Elizabeth Gargon, Paula R. Williamson, on behalf of the COS-STAR Group

**Affiliations:** Department of Biostatistics, University of Liverpool, Liverpool, UK; Centre for Statistics in Medicine, University of Oxford, Oxford, UK; School of Social & Community Medicine, University of Bristol, Bristol, UK; Centre for Public Health, Queen’s University Belfast, Belfast, UK; National University of Ireland Galway & West Northwest Hospitals Group, Galway, Ireland

**Keywords:** COMET, Core outcome set, COS-STAR, Outcomes, Reporting guideline

## Abstract

**Background:**

Core outcome sets can increase the efficiency and value of research and, as a result, there are an increasing number of studies looking to develop core outcome sets (COS). However, the credibility of a COS depends on both the use of sound methodology in its development and clear and transparent reporting of the processes adopted. To date there is no reporting guideline for reporting COS studies. The aim of this programme of research is to develop a reporting guideline for studies developing COS and to highlight some of the important methodological considerations in the process.

**Methods/Design:**

The study will include a reporting guideline item generation stage which will then be used in a Delphi study. The Delphi study is anticipated to include two rounds. The first round will ask stakeholders to score the items listed and to add any new items they think are relevant. In the second round of the process, participants will be shown the distribution of scores for all stakeholder groups separately and asked to re-score. A final consensus meeting will be held with an expert panel and stakeholder representatives to review the guideline item list. Following the consensus meeting, a reporting guideline will be drafted and review and testing will be undertaken until the guideline is finalised. The final outcome will be the COS-STAR (Core Outcome Set-STAndards for Reporting) guideline for studies developing COS and a supporting explanatory document.

**Discussion:**

To assess the credibility and usefulness of a COS, readers of a COS development report need complete, clear and transparent information on its methodology and proposed core set of outcomes. The COS-STAR guideline will potentially benefit all stakeholders in COS development: COS developers, COS users, e.g. trialists and systematic reviewers, journal editors, policy-makers and patient groups.

## Background

Clinical trials seek to evaluate whether an intervention is effective by comparing the effects of interventions on outcomes chosen to identify benefits and harms. The selection of appropriate outcome domains and measures within those domains is crucial to the design of trials. Outcomes need to be relevant to health service users and other people making choices about health care if the findings of research are to influence practice and future research. There is a growing recognition that insufficient attention has been paid to the outcomes measured in clinical trials.

A core outcome set (COS) is an agreed set of outcomes that should be measured and reported, as a minimum, in all clinical trials in specific areas of health or health care [1]. COS could have implications across all areas of research in health and health and social care, reduce heterogeneity between outcomes reported in trials, lead to research that is more likely to have measured relevant outcomes, and enhance the value of evidence synthesis in the future by reducing the risk of outcome reporting bias and ensuring that all trials contribute usable information to similar important outcomes.

To help address these problems, the Core Outcome Measures in Effectiveness Trials (COMET) Initiative, www.comet-initiative.org, was launched in 2010, and has four main objectives:

1. To raise awareness of current problems with outcomes in clinical trials.
2. To encourage COS development and uptake.
3. To provide resources to allow practitioners to develop COS, e.g. the COMET database of existing and ongoing COS studies.
4. To encourage evidence-based COS development.

The COMET database currently contains 261 papers related to 207 published COS. The credibility of a COS depends on both the use of sound methodology in its development and clear and transparent reporting of the processes adopted. Although the issues that groups should consider when developing a COS have recently been described, to date there is no formal COS reporting guideline available [2].

The aim of this research project is to develop a guideline (COS-STAR, Core Outcome Set-STAndards for Reporting) for the reporting of studies developing COS and an explanatory document using the approach proposed by EQUATOR (Enhancing the QUAlity and Transparency Of health Research) [3].

### An explanation of the terms used in this proposal

#### Steering Committee membership

The five-member panel Paula Williamson (Director of the MRC North West Hub for Trials Methodology Research), Doug Altman (Executive of the CONSORT Group and Chair of the EQUATOR Network), Jane Blazeby (Director of the MRC ConDuCT-II Hubs for Trials Methodology Research (HTMR)), Mike Clarke (Director of the MRC All-Ireland HTMR) and Elizabeth Gargon (Project Co-ordinator for the COMET Initiative) form the COMET Management Group (COMET MG).

For the purposes of the COS reporting guideline development project two additional members will join the research team: Jamie Kirkham (Co-Lead Hub for Trials Methodology Research Outcomes Working Group), Sarah Gorst (researcher into core outcome sets) and Declan Devane (Director, Trials Methodology Research Network, Ireland). The 8 team members combined make up the Steering Committee*.*

#### Expert panel

An expert panel of five members has been convened to review and guide the Steering Committee at each stage of the project. Members of this panel include Peter Tugwell (OMERACT (Outcome Measures in Rheumatology) Executive Committee), Rosalind Raine (Director of National Institute for Health Research (NIHR) Collaboration for Leadership in Applied Health Research and Care (CLAHRC)), David Moher (author of reporting guidelines), Jochen Schmitt (Harmonising Outcome Measures for Eczema (HOME) Initiative) and Sean Tunis (Director of the Centre for Medical Technology Policy).

#### Delphi participants

Participants in the Delphi survey.

#### Consensus meeting participants

Participants attending the consensus meeting.

## Methods/Design

The development of the COS guideline will be undertaken in five stages:

1. Establish a preliminary checklist of reporting items to be considered for inclusion in the COS reporting guideline (Stage 1).
2. Conduct a Delphi survey to gain consensus opinion on reporting items to be considered within a standardised reporting guideline for COS development studies (Stage 2).
3. Hold a consensus meeting to identify the main items to be included in the definitive reporting guideline for COS development studies (Stage 3).
4. Develop a high-quality reporting guideline and a detailed explanatory document (Stage 4).
5. Post-development activities: pilot-testing and dissemination (Stage 5).

The role of the Steering Committee will be to:

1. Review and finalise the proposed research protocol.
2. Identify the preliminary checklist of reporting items.
3. Monitor and review the results of each round of the Delphi.
4. Attend and help facilitate the consensus meeting.
5. Review, finalise and contribute to the publication and dissemination of the reporting guideline and the explanatory document.

### Stage 1

#### Preliminary checklist of reporting items

Based on previous discussions among the COMET MG, a published preliminary checklist of items that groups should consider when reporting the development of a COS already exists [2, 4]. Through discussion and personal experience with COS development, the Steering Group members will supplement this list with any additional items thought to be important. The Steering Group will approve this extended list as the initial list of reporting items for COS developers.

### Stage 2

#### Delphi survey

## Design

The Delphi process will consist of two rounds of electronic-based questionnaire, response and feedback. The first-round survey will include scoring of reporting guideline items informed by the preliminary checklist from Stage 1 and invite additional items not included in the preliminary list. A second round will then be undertaken providing feedback from the previous round and inviting further response from participants. Any additional reporting items identified by participants in Round 1 will be included for consideration in the second round of the Delphi process.

## Participants

Four particular stakeholder groups will be invited to participate. Stakeholder groups were chosen to encompass all aspects of COS development. Firstly those that develop COS (COS developers), those that publish the COS articles (editors), users of COS (trialists, systematic reviewers and clinical guideline developers) and, finally, a patient group.

(i) COS developers (those who have been involved in at least 1 published COS, both clinical and methodological leads) (approximately 200 lead investigators from the COMET database, 16 February 2015)
(ii)  Editors of journals:
  - Journal editors where COS studies have been published (approximately 250 unique journals from COMET database, 16 February 2015)
  - CROWN (CoRe Outcomes in WomeN’s health) journal editors (approximately 70 journals, 10 March 2015)
(iii)   COS users:
  - Trialists – principal investigators or co-investigators of open phase III/IV trials registered on Clinical Trial.gov (an approximate 20 % random sample from 8449 registered trials, last verified April 2015)
  - Systematic reviewers: Cochrane Review Group co-ordinating editors (76 co-ordinating editors from 53 review groups)
  - Clinical guideline developers (e.g. NICE (National Institute for Health and Care Excellence) (currently nearly 100 organisations listed on the Guidelines International Network website, http://www.gin2015.net/about/, 10 March 2015)
(iv)   Patient facilitators of patient and public involvement (PPI) workshops and members of the COMET Public and Patient Participation, Involvement and Engagement (PoPPIE) Working Group (approximately 30 members)

The expert panel members have experience in many of these roles and will also be invited to take part in the Delphi exercise.

## Recruitment process

Participants will be sent a personalised Email outlining the project, and will be asked to complete Round 1 of the Delphi exercise within 3 weeks. Contact authors from the COS developers stakeholder group will be asked to forward the Delphi survey onto their co-authors. This snowballing technique will ensure that we obtain the opinions of both clinical and methodological experts in COS development. A reminder Email will be sent at the end of week 2 to prompt completion of the survey. Participation into the survey is optional and informed consent will be assumed if a participant responds to the survey.

All participants will be allocated a unique identification number to allow the identification of individual responses in both rounds of the Delphi exercise. Demographic data regarding the participant’s profession, previous involvement with COS development and reporting guideline development will be collected. Participants will be invited to provide their name and consent to be acknowledged as a member of the Delphi panel in the publication arising from this research. All participants who complete the first round of the Delphi will be invited to participate in the second.

## Delphi scoring

Participants will be asked to score each of the reporting guideline items listed using a scale of 1 to 9, with 1 to 3 labelled ‘not important for inclusion in the guideline’, 4 to 6 labelled ‘important but not critical for inclusion in the guideline’ and 7 to 9 labelled ‘critical for inclusion into the guideline’ [5]. Participants will also be allowed to score ‘unsure’ if they are unable to offer an opinion as to whether the reporting guideline item is important or not.

## Software

The Delphi process will be conducted using an electronic bespoke survey format which has been previously developed by the COMET Initiative for use in COS development projects.

## Delphi Round 1

Reporting guideline items will be presented in chronological order to reflect the stage and topic of the reporting items (e.g. title, abstract, introduction, methods, results, discussion and editorial independence) [2]. Participants will be asked to score each item. After completing the rating exercise, participants will have the opportunity to add any other items that they believe should be added when reporting studies developing COS.

## First round analysis

Additional guideline reporting items listed by participants will be reviewed by the research team to ensure they represent a new item. For each item, the number of participants who have scored the item and the distribution of scores will be summarised. All items will be carried forward to Round 2.

The participant response rate will be calculated as the total number of respondents who completed the survey as a percentage of those to whom an Email invitation was sent. The number of participants that accessed the survey but did not complete it will also be monitored.

## Delphi Round 2

In Round 2, each participant who participated in Round 1 will be shown the number of respondents and distribution of scores for each item, for all stakeholder groups separately with their own score from Round 1. They will be asked to consider the responses from other Delphi participants, and to re-score the item. In addition, if a participant changed their score from not critical (<7 in Round 1) to critical in Round 2 (7 or more) or from critical in Round 1 to not critical in Round 2, they will be asked to provide the reason for this change.

## Second round analysis

For each item, the number of participants who have scored it and the distribution of scores will be summarised. The number of participants completing Round 2 will be documented and the potential for attrition bias will be assessed by comparing the participant scores for those who completed both rounds with those who completed Round 1 only. We will also examine changes in participant scores between rounds and summarise the reasons given for these changes.

## Consensus definition

Consensus will be defined ‘a priori’, as previously recommended [4]. Guideline reporting items will be prioritised if they gain the support from at least 70 % of participants scoring ‘critical’, i.e. score 7 to 9.

## Statistical considerations

The approximate sample sizes listed in the participation section above are sufficiently large to yield a meaningful statistical analysis even after non-responses/attrition is taken into consideration. Given the design, in which results from each stakeholder group will be summarised separately, we will take the approach of trying to maximise the information, within practical limitations. Efforts will be taken to maximise the response rate across stakeholder groups.

### Stage 3

#### Consensus meeting

In addition to the Steering Committee and expert panel members, further representatives of the stakeholder groups included in the Delphi exercise will be invited to a 2-day face-to-face consensus meeting of approximately 20 people. The places available outside the Steering Committee and expert panel will be chosen such that all stakeholder groups are reasonably represented. The format of the consensus meeting will comprise a short study overview, a presentation containing a summary of the results of how each stakeholder group had scored each reporting guideline item (from Stage 2), and the number of stakeholder groups who achieved consensus. Reporting guideline items that reached consensus from all stakeholder groups will be considered first in order to ratify those results. Each remaining item will then be considered in turn according to the number of stakeholder groups where consensus was achieved, i.e. the next batch of items to be considered will be those that reached consensus in all but one stakeholder group. All consensus meeting participants will be given an opportunity to discuss each item (if they wish). Discussion of each reporting item will then be followed by an anonymous scoring method by those at the consensus meeting. The final detail of the agenda, structure and content of the meeting will be set by the Steering Committee based on a review of the responses from Delphi participants. Members of the Steering Committee will chair the relevant sessions as appropriate.

The main goal of the consensus meeting will be to identify the items that will be included in the reporting guideline for COS development studies. Similar to the Delphi stage, reporting guideline items will be considered as consensus in, if 70 % of the consensus meeting participants vote in favour of the item to be included in the guideline. The meeting will also agree on publication and dissemination strategies. The aim of this specific discussion will be to determine the best form of communication, methods of feedback (e.g. track changes), to provide a structure and work plan for writing of the draft guideline and methods for resolving disagreements. Following the consensus meeting, a written report on the outcome of the meeting will be circulated to the consensus meeting participants for comment.

### Stage 4

#### Development of reporting guideline and explanatory document

The purpose of the explanatory document is to provide the background, rationale and justification for the COS reporting guideline as well as provide examples for the users regarding what information should be included and how this should be reported. The explanatory document will be developed concurrently with the reporting guideline.

## Procedure

Following the discussions on dissemination strategies with the consensus meeting participants (Stage 3), the Steering Committee will draft the initial guideline for reporting studies developing COS and the explanatory document. The basis of each reporting item will be described in the draft recommendations. This will include the origin of the item (Steering Committee or Delphi), the degree of the consensus achieved (Delphi and consensus meeting) and a brief rationale for inclusion supplemented with examples of good and bad reporting practices.

The draft document will be provided to the expert panel who will be given an opportunity to provide comment on the guideline and explanatory document. The COS guideline will undergo subsequent review and revisions by the research team and expert panel until a final draft is considered complete. Consideration will be made on the content, layout and wording of the document.

### Stage 5

#### Pilot-testing of the reporting guideline

In the final stage, we will pilot-test the draft reporting guideline with both COS developers who are looking to write-up the findings to their ongoing COS project and those who have already published a COS project. The COMET database has identified 65 unpublished COS development projects. Lead authors will be invited to test the guideline as they write-up their COS study. More experienced COS developers would be asked to review the guideline against their published report to see if the guideline items make sense and would have improved their reporting. Their feedback on the content, format, and usefulness of the guideline will be obtained through an anonymous survey and incorporated into the final COS reporting guideline.

## Ethics

The University of Liverpool Ethics Committee has been consulted and confirmed Ethical approval for this study (Reference RETH000841).

## Publication plan

- Publication 1: Study protocol
- Publication 2: COS reporting guideline statement
- Publication 3: COS reporting guideline explanatory document

## Discussion

There is currently no guideline for the reporting of studies developing COS that has been developed using a widely-accepted robust methodology. With the increasing number of COS being developed, this guideline will not only improve the transparency of reporting of studies that are developing COS but will also highlight some of the important methodological considerations. The guideline will potentially benefit all stakeholders in COS development: COS developers, COS users, e.g. trialists and systematic reviewers, journal editors, policy- makers and patient groups. As of August 2015, the preliminary checklist of reporting items has been drawn up and approved by the Steering Group (Stage 1). The Delphi system has also been developed and participant lists have been put together for each stakeholder group. Currently the Delphi is live and is receiving responses for Round 1.

## Abbreviations

CLAHRC: Collaboration for Leadership in Applied Health Research and Care
COMET: Core Outcome Measures in Effectiveness Trials
COS: core outcome set
COS-STAR: Core Outcome Set-STAndards for Reporting
CROWN: CoRe Outcomes in WomeN’s health
EQUATOR: Enhancing the QUAlity and Transparency Of health Research
HOME: Harmonising Outcome Measures for Eczema
HTMR: Hubs for Trials Methodology Research
NIHR: National Institute for Health Research
OMERACT: Outcome Measures in Rheumatology
PoPPIE: Public and Patient Participation, Involvement and Engagement
PPI: patient and public involvement
